# Applied Microbiology and Biotechnology: Challenges in Food Microbiology and Food Safety Research

**DOI:** 10.3390/foods15142453

**Published:** 2026-07-10

**Authors:** Aleksandra Ranitović

**Affiliations:** Faculty of Technology Novi Sad, University of Novi Sad, Bulevar cara Lazara 1, 21000 Novi Sad, Serbia; a.ranitovic@uns.ac.rs

Food microbiology is an increasingly interdisciplinary field, essential to modern global food systems. It covers topics from ensuring food safety and preventing spoilage to the use of microorganisms for industrial-scale biotechnology and the development of functional foods. Recent advances in molecular biology, microbial ecology, and biotechnology have significantly expanded the role of food microbiology from traditional pathogen detection to integrated approaches that support food quality, sustainability, and functionality [1,2]. The Special Issue entitled “Challenges in Food Microbiology and Food Safety Research” was launched to present recent advances in addressing key challenges in food safety and quality, microbial biotechnology, and functional food development.

With the expansion of global food trade and increasingly complex food supply chains, ensuring food safety and authenticity has become increasingly challenging. Foodborne diseases caused by pathogenic microorganisms continue to pose a risk to public health worldwide, while food fraud and quality degradation are growing concerns for consumers and manufacturers. Traditional microbiological and analytical methods have played a notable role in food quality control, but are often limited by long analysis times, labor-intensive procedures, and insufficient sensitivity [3,4,5]. Consequently, food microbiology has witnessed a rapid transition toward molecular and omics-based analytical approaches, including qPCR, LAMP, DNA barcoding, genomics, proteomics, and metabolomics. These approaches offer improved sensitivity, specificity, and speed, thus enabling more efficient detection of foodborne pathogens, spoilage-causing microorganisms, and markers of authenticity.

Another important trend shaping food microbiology is the growing interest in functional foods and beverages. Growing consumer awareness of the link between nutrition and health has fueled demand for products that provide benefits beyond a basic diet [6]. Fermented foods and beverages containing probiotic microorganisms have gained considerable attention due to their potential health benefits. However, maintaining microbial viability during processing, storage, and gastrointestinal transit remains a major technological challenge. Consequently, microencapsulation and related protective technologies are increasingly explored to improve probiotic stability and functionality [7,8].

Sustainability has also become a focus of food microbiology and biotechnology. The shift to more sustainable food production systems has sparked interest in using microorganisms as efficient platforms for producing high-value compounds, including enzymes, bioactive molecules, lipids, proteins, and other industrially relevant products. In this context, white biotechnology strives to develop environmentally friendly production processes that reduce reliance on non-renewable resources, while enabling the valorization of agricultural and industrial by-products [9].

An important theme emerging from this Special Issue is the growing need for rapid, reliable molecular tools for food safety monitoring and authenticity assessment. Three contributions focused on nucleic acid-based technologies and their applications in food systems. These studies highlighted the potential of advanced molecular methods for detecting spoilage microorganisms and foodborne pathogens, as well as for authenticating food products. Together, they demonstrate how techniques such as quantitative real-time polymerase chain reaction (qPCR) [Contribution 1], colorimetric loop-mediated isothermal amplification (LAMP) [Contribution 2], and DNA barcoding [Contribution 3] can improve the speed, sensitivity, and accuracy of food quality control while supporting traceability and consumer protection.

Another important contribution addressed the development of functional foods by incorporating beneficial microorganisms. The study investigating kombucha fortified with whey protein-encapsulated lactic acid bacteria demonstrated how encapsulation technologies can be used to enhance microbial stability while simultaneously influencing the functional properties of fermented beverages [Contribution 4]. Such approaches contribute to the growing body of research focused on improving the health-promoting potential and technological robustness of functional food products.

The review dedicated to the genus *Yarrowia* provided a comprehensive overview of its taxonomy, physiology, ecology, and biotechnological potential. The review emphasized the importance of microbial platforms for developing sustainable bioprocesses and value-added products [Contribution 5].

Collectively, the contributions published in this Special Issue demonstrate how food microbiology is evolving beyond its traditional focus on pathogen detection and food preservation toward an increasingly interdisciplinary field that integrates molecular diagnostics, functional microorganisms, and sustainable biotechnology. These studies together illustrate how advances in analytical technologies, microbial applications, and bioprocesses can simultaneously enhance food safety, food authenticity, product functionality, and sustainability. At the same time, they identify several priorities for future research, including the continued development of rapid molecular diagnostic tools, innovative strategies for improving the stability and functionality of probiotic microorganisms, and the expansion of sustainable microbial platforms for industrial food biotechnology. These directions highlight the growing role of food microbiology as a key driver of innovation within modern food systems.

Building upon the advances highlighted in this Special Issue, several priorities are expected to shape future research in applied food microbiology and biotechnology. One of the most promising directions involves the further development of rapid, sensitive, and portable diagnostic technologies for food safety monitoring. Future efforts should focus on improving method standardization, reducing costs, and facilitating large-scale implementation within routine food safety monitoring programs. The development of functional foods is also expected to remain an important research priority. Attention should be given to technologies that enhance probiotic viability and stability throughout processing, storage, and gastrointestinal transit. Additionally, a deeper understanding of interactions between food matrices, probiotic microorganisms, and the human gut microbiome may support the development of more effective and personalized functional food products. Sustainability considerations will continue to drive innovation in microbial biotechnology. Future research should focus on strain improvement, metabolic engineering, process optimization, and the utilization of renewable industrial by-products to support circular bioeconomy principles and environmentally sustainable production systems.

Another important trend is the integration of artificial intelligence, machine learning, and predictive modeling into food microbiology research. These tools may facilitate risk assessment, process optimization, microbial behavior prediction, and data-driven decision-making across food production chains.

Overall, the future of food microbiology will rely on integrating advanced analytical technologies, sustainable bioprocesses, and interdisciplinary collaboration. The contributions presented in this Special Issue not only address current challenges but also provide valuable perspectives for future research aimed at improving food safety and quality, and at developing innovative and sustainable food systems.
